# Supplementary material for: RAS mutations drive proliferative chronic myelomonocytic leukemia via a KMT2A-PLK1 axis
Source: Nat Commun. 2021 May 18;12:2901. doi: 10.1038/s41467-021-23186-w (PMC8131698; doi:10.1038/s41467-021-23186-w)
Supplement: Supplementary file 1 — Supplementary Information [file 41467_2021_23186_MOESM1_ESM.pdf]

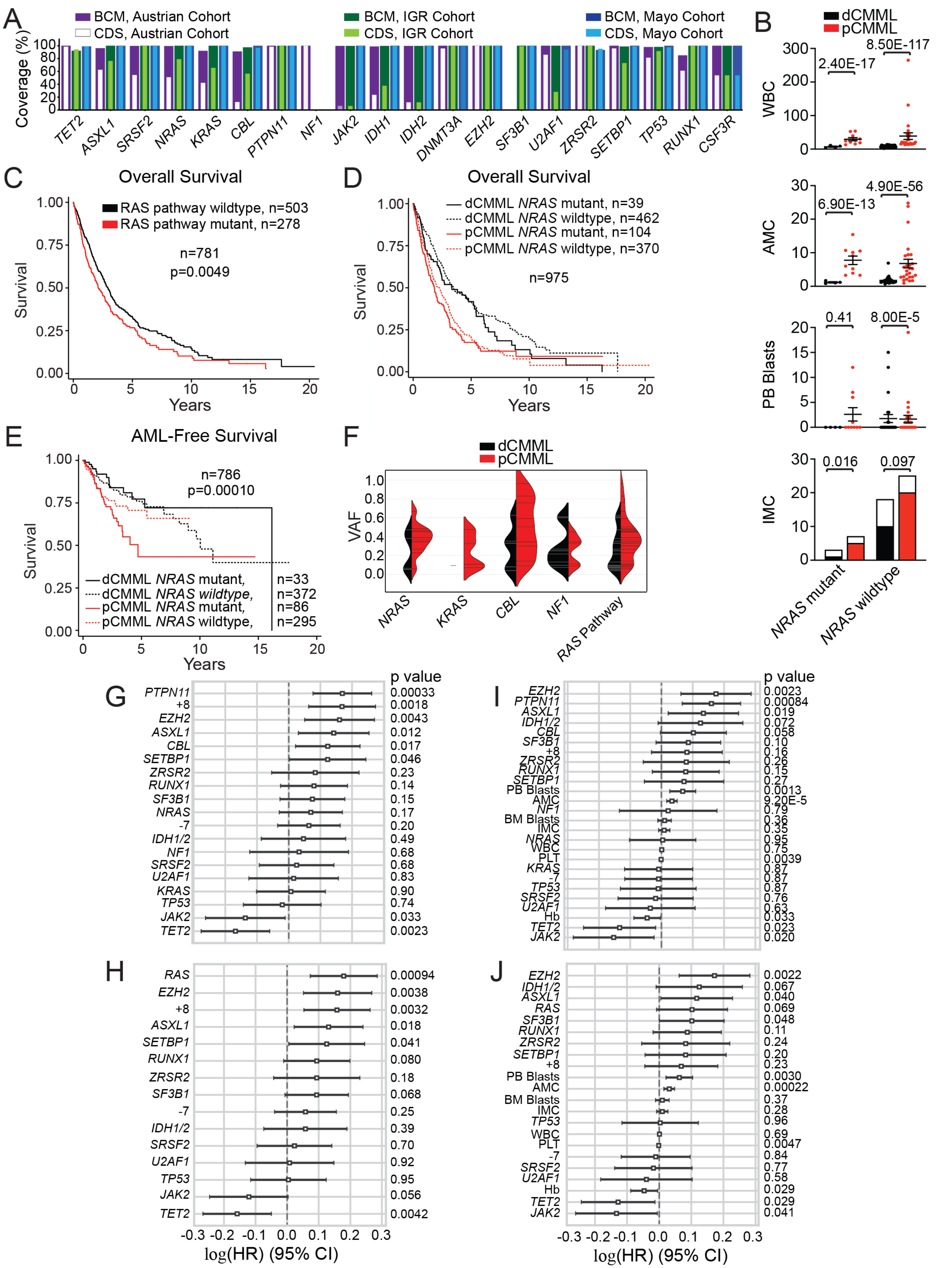

A
BCM, Austrian Cohort
CDS, Austrian Cohort
BCM, IGR Cohort
CDS, IGR Cohort
BCM, Mayo Cohort
CDS, Mayo Cohort
Coverage (%)
TET2 ASXL1 SRSF2 NRAS KRAS CBL PTPN11 NF1 JAK2 IDH1 IDH2 DNMT3A EZH2 SF3B1 U2AF1 ZRSR2 SETBP1 TP53 RUNX1 CSF3R
B
dCMML
pCMML
WBC
2.40E-17
8.50E-117
AMC
6.90E-13
4.90E-56
PB Blasts
0.41
8.00E-5
IMC
0.016
0.097
NRAS mutant
NRAS wildtype
C
Overall Survival
RAS pathway wildtype, n=503
RAS pathway mutant, n=278
n=781
p=0.0049
Survival
Years
D
Overall Survival
dCMML NRAS mutant, n=39
dCMML NRAS wildtype, n=462
pCMML NRAS mutant, n=104
pCMML NRAS wildtype, n=370
n=975
Survival
Years
E
AML-Free Survival
n=786
p=0.00010
dCMML NRAS mutant, n=33
dCMML NRAS wildtype, n=372
pCMML NRAS mutant, n=86
pCMML NRAS wildtype, n=295
Survival
Years
F
dCMML
pCMML
VAF
NRAS KRAS CBL NF1 RAS Pathway
G
p value
PTPN11 0.00033
+8 0.0018
EZH2 0.0043
ASXL1 0.012
CBL 0.017
SETBP1 0.046
ZRSR2 0.23
RUNX1 0.14
SF3B1 0.15
NRAS 0.17
-7 0.20
IDH1/2 0.49
NF1 0.68
SRSF2 0.68
U2AF1 0.83
KRAS 0.90
TP53 0.74
JAK2 0.033
TET2 0.0023
H
RAS 0.00094
EZH2 0.0038
+8 0.0032
ASXL1 0.018
SETBP1 0.041
RUNX1 0.080
ZRSR2 0.18
SF3B1 0.068
-7 0.25
IDH1/2 0.39
SRSF2 0.70
U2AF1 0.92
TP53 0.95
JAK2 0.056
TET2 0.0042
log(HR) (95% CI)
I
p value
EZH2 0.0023
PTPN11 0.00084
ASXL1 0.019
IDH1/2 0.072
CBL 0.058
SF3B1 0.10
+8 0.16
ZRSR2 0.26
RUNX1 0.15
SETBP1 0.27
PB Blasts 0.0013
AMC 9.20E-5
NF1 0.79
BM Blasts 0.36
IMC 0.35
NRAS 0.95
WBC 0.75
PLT 0.0039
KRAS 0.87
-7 0.87
TP53 0.87
SRSF2 0.76
U2AF1 0.63
Hb 0.033
TET2 0.023
JAK2 0.020
J
EZH2 0.0022
IDH1/2 0.067
ASXL1 0.040
RAS 0.069
SF3B1 0.048
RUNX1 0.11
ZRSR2 0.24
SETBP1 0.20
+8 0.23
PB Blasts 0.0030
AMC 0.00022
BM Blasts 0.37
IMC 0.28
TP53 0.96
WBC 0.69
PLT 0.0047
-7 0.84
SRSF2 0.77
U2AF1 0.58
Hb 0.029
TET2 0.029
JAK2 0.041
log(HR) (95% CI)

Supplementary Figure 1. RAS pathway mutations are associated with poor outcomes and are enriched in pCMML. A. Gene and respective exon coverage of next generation sequencing panels used. BCM = Blood Cancer Mutations, CDS = Coding Sequences. B. Assessment of four clinical parameters including white blood cell (WBC) count, absolute monocyte count (AMC), peripheral blood (PB) blasts, and circulating immature myeloid cells (IMC) in *NRAS* mutant dCMML compared with *NRAS* mutant pCMML (n=4 vs 10, left) and in *NRAS* wildtype dCMML compared with *NRAS* wildtype pCMML (n=24 vs n=27, right). The indicated n represents number of patients. Indicated p-values are by Mann-Wittney test. C. Kaplan-Meier curve depicting survival in CMML stratified based on RAS pathway mutation status. Indicated p-value by log-rank test. D. Kaplan-Meier curve depicting overall survival of CMML patients stratified by *NRAS* mutation status. E. Kaplan-Meier curve depicting AML-free survival of CMML patients stratified by *NRAS* mutation status. Indicated p-value by log-rank test. F. Violin plots representing variant allele frequencies (VAFs) of *NRAS*, *KRAS*, *CBL*, *NF1* and RAS pathway mutations in dCMML and pCMML. VAF is depicted on the y-axis. Width of horizontal hatches correlates to number of samples with the indicated VAF. G-J. Multivariable models to predict survival related to driver mutations alone (G and H, n=781 individual patients) and driver mutations in the context of clinical variables (I and J, n=744 individual patients). X-axis represents the natural logarithm of hazard ratios (HR). Bars represent confidence intervals (CI). Indicated p-values in panels G-J by log likelihood ratio test. The mean is the measure of center. Data in panel B are presented as mean ± SEM. Source data are provided as a Source Data file.

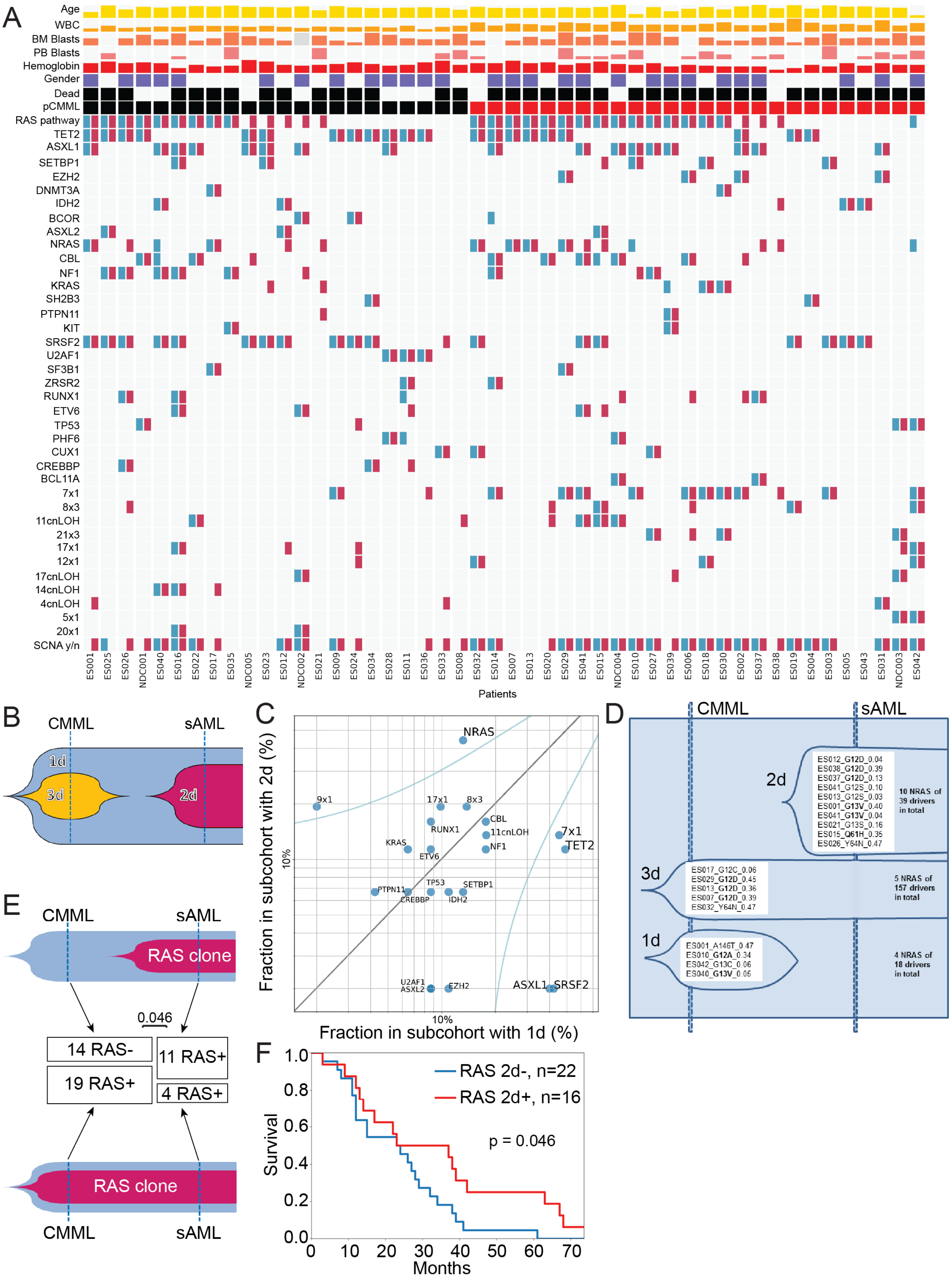
A
Age
WBC
BM Blasts
PB Blasts
Hemoglobin
Gender
Dead
pCMML
RAS pathway
TET2
ASXL1
SETBP1
EZH2
DNMT3A
IDH2
BCOR
ASXL2
NRAS
CBL
NF1
KRAS
SH2B3
PTPN11
KIT
SRSF2
U2AF1
SF3B1
ZRSR2
RUNX1
ETV6
TP53
PHF6
CUX1
CREBBP
BCL11A
7x1
8x3
11cnLOH
21x3
17x1
12x1
17cnLOH
14cnLOH
4cnLOH
5x1
20x1
SCNA y/n
ES001 ES025 ES026 NDC001 ES040 ES016 ES022 ES017 ES035 NDC005 ES023 ES012 NDC002 ES021 ES009 ES024 ES034 ES028 ES011 ES036 ES033 ES008 ES032 ES014 ES007 ES013 ES020 ES029 ES041 ES015 NDC004 ES010 ES027 ES039 ES006 ES018 ES030 ES002 ES037 ES038 ES019 ES004 ES003 ES005 ES043 ES031 NDC003 ES042
Patients
B
CMML
sAML
1d
3d
2d
C
NRAS
9x1
17x1
8x3
RUNX1
CBL
11cnLOH
7x1
KRAS
NF1
TET2
ETV6
TP53
SETBP1
PTPN11
CREBBP
IDH2
U2AF1
ASXL2
EZH2
ASXL1
SRSF2
10%
10%
Fraction in subcohort with 2d (%)
Fraction in subcohort with 1d (%)
D
CMML
sAML
2d
ES012_G12D_0.04
ES038_G12D_0.39
ES037_G12D_0.13
ES041_G12S_0.10
ES013_G12S_0.03
ES001_G13V_0.40
ES041_G13V_0.04
ES021_G13S_0.16
ES015_Q61H_0.35
ES026_Y64N_0.47
10 NRAS of 39 drivers in total
3d
ES017_G12C_0.06
ES029_G12D_0.45
ES013_G12D_0.36
ES007_G12D_0.39
ES032_Y64N_0.47
5 NRAS of 157 drivers in total
1d
ES001_A146T_0.47
ES010_G12A_0.34
ES042_G13C_0.06
ES040_G13V_0.05
4 NRAS of 18 drivers in total
E
CMML
sAML
RAS clone
0.046
14 RAS-
11 RAS+
19 RAS+
4 RAS+
RAS clone
CMML
sAML
F
RAS 2d-, n=22
RAS 2d+, n=16
p = 0.046
Survival
Months
1.0
0.8
0.6
0.4
0.2
0.0
0
10
20
30
40
50
60
70

Supplementary Figure 2. Driver mutation alterations encountered in CMML progression to AML. A. Driver mutations and somatic copy number alterations (SCNAs) in CMML patients that progressed to sAML. For each patient (individual columns) putative driver events at CMML (blue) and sAML (red) are depicted. Clinical characteristics correspond to measurements at the CMML stage. B. Categorization of mutations detected in CMML and sAML sample pairs. 1d are mutations detected in both CMML and sAML and considered as primary drivers, 2d are those detected only in sAML and considered secondary drivers, and 3d are those detected in CMML and lost in sAML, suggesting subclonal secondary drivers. C. Prevalence of the main driver mutations and SCNAs in the categories of primary driver (x-axis) events (1d) and secondary driver events (2d), occurring during leukemic transformation (LT) (y-axis). Confidence intervals represent Fisher exact test alpha = 0.05 for point mutations. D. Fraction of *NRAS* mutations among all drivers in CMML (1d), gained (2d) or lost (3d) during LT. E. Diagrams of CMML with (below) and without (above) RAS-driven subclones. CMML without *RAS* mutations are at increased risk for acquisition of *RAS* mutations during LT. Indicated p-value by one-tailed Fisher's exact test. F. Kaplan-Meier curve depicting overall survival of CMML with and without *RAS* mutations during LT. The indicated n represents the number of patients. Indicated p-value by log-rank test.

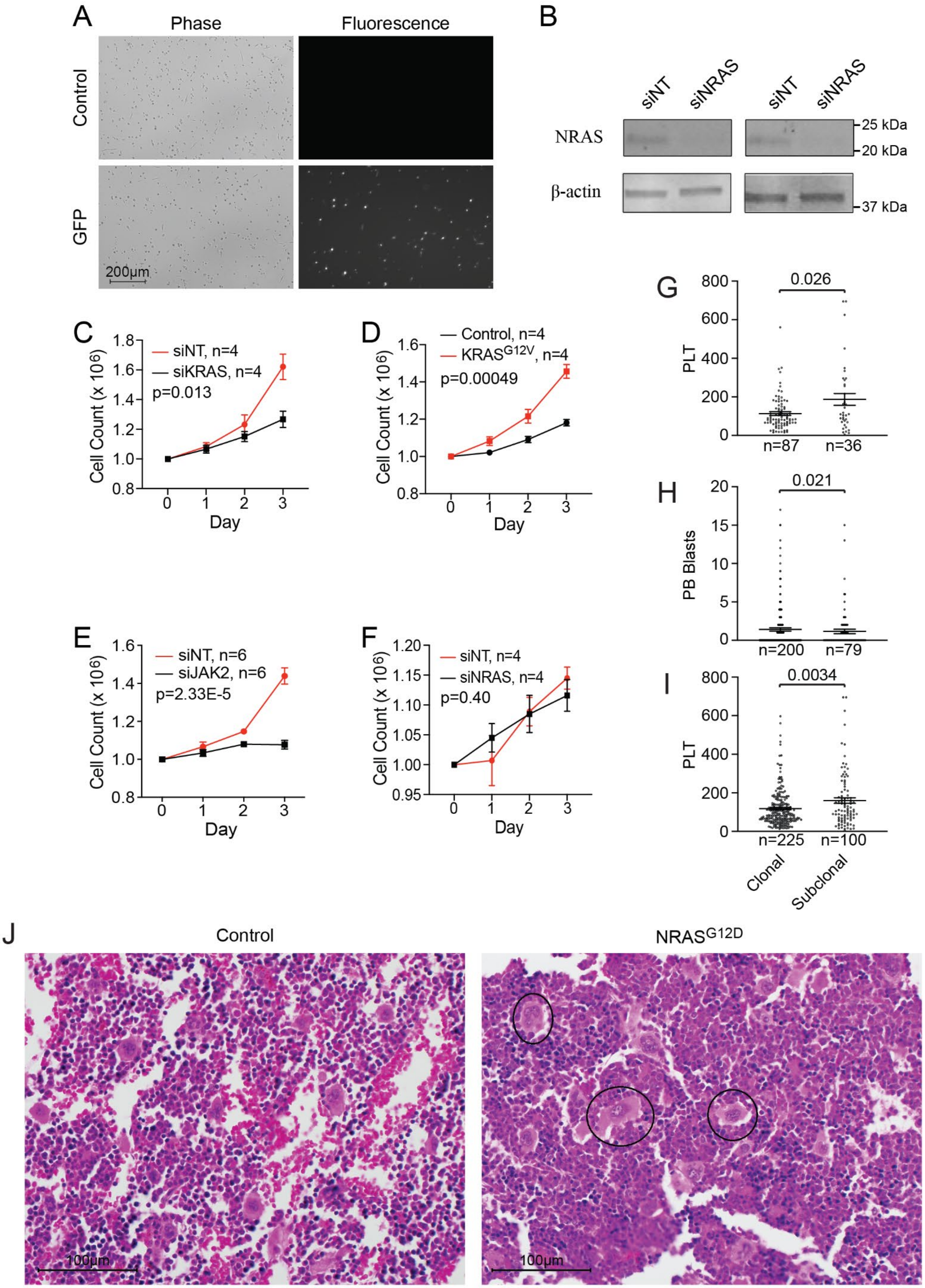

A
Phase
Fluorescence
Control
GFP
200μm
B
siNT
siNRAS
siNT
siNRAS
NRAS
β-actin
25 kDa
20 kDa
37 kDa
C
siNT, n=4
siKRAS, n=4
p=0.013
Cell Count (x 10^6)
Day
D
Control, n=4
KRAS^G12V, n=4
p=0.00049
Cell Count (x 10^6)
Day
E
siNT, n=6
siJAK2, n=6
p=2.33E-5
Cell Count (x 10^6)
Day
F
siNT, n=4
siNRAS, n=4
p=0.40
Cell Count (x 10^6)
Day
G
0.026
PLT
n=87
n=36
H
0.021
PB Blasts
n=200
n=79
I
0.0034
PLT
n=225
n=100
Clonal
Subclonal
J
Control
NRAS^G12D
100μm
100μm

Supplementary Figure 3. Effect of drivers NRAS, KRAS and JAK2 on the CMML proliferative phenotype. A. Representative phase (left) and fluorescent (right) microscopy images of CMML patient-derived MNCs after electroporation of either an empty vector (Control, above) or GFP expression construct (below). B. Representative Western blots demonstrating efficiency of NRAS knockdown after electroporation of CMML patient-derived MNCs with either a non-target siRNA (siNT) or siRNA against NRAS (siNRAS). These are representative of three experiments. C-F. Daily cell counts of CMML patient-derived MNCs after siRNA depletion of KRAS in *KRAS* mutant pCMML cells (C), overexpression of KRASG12V in *KRAS* wildtype dCMML cells (D), siRNA depletion of JAK2 in *JAK2* mutant pCMML cells (E), and siRNA depletion of NRAS in *NRAS* mutant dCMML cells (F). Indicated p-values for panels C-F are by two-tailed Student's t-test. G. Platelet counts (PLT) in CMML with clonal (n=87) and subclonal (n=37) VAF_{NRAS}. Threshold VAF = 0.29. H and I. PB blasts (H) and PLT (I) in CMML with clonal (n=230) and subclonal (n=106) VAF of five RAS genes. Threshold VAF = 0.29. Indicated p-values for panels G-I are by Mann-Wittney test. J. Enlarged H&E bone marrow slides at 400X magnification from control (left) and $Nras^{G12D}$ (right) mice. Dysplastic megakaryocytes are indicated (circles). Data in panels C-I are presented as mean ± SEM. The indicated n represents the number of biologic replicates. Source data are provided as a Source Data file.

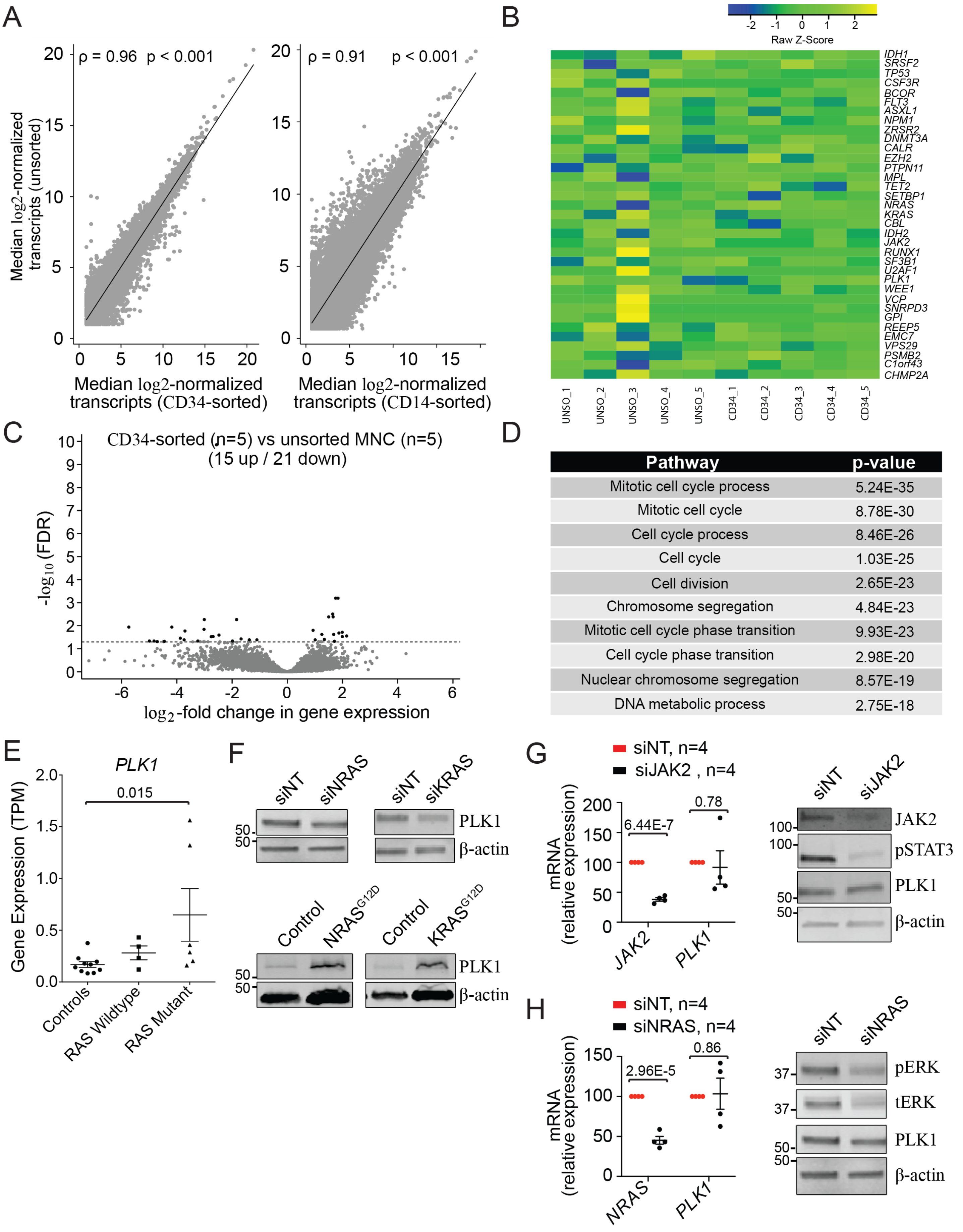

A
ρ = 0.96 p < 0.001
ρ = 0.91 p < 0.001
Median log2-normalized transcripts (unsorted)
Median log2-normalized transcripts (CD34-sorted)
Median log2-normalized transcripts (CD14-sorted)
B
Raw Z-Score
IDH1
SRSF2
TP53
CSF3R
BCOR
FLT3
ASXL1
NPM1
ZRSR2
DNMT3A
CALR
EZH2
PTPN11
MPL
TET2
SETBP1
NRAS
KRAS
CBL
IDH2
JAK2
RUNX1
SF3B1
U2AF1
PLK1
WEE1
VCP
SNRPD3
GPI
REEP5
EMC7
VPS29
PSMB2
C1orf43
CHMP2A
UNSO_1
UNSO_2
UNSO_3
UNSO_4
UNSO_5
CD34_1
CD34_2
CD34_3
CD34_4
CD34_5
C
CD34-sorted (n=5) vs unsorted MNC (n=5)
(15 up / 21 down)
-log10 (FDR)
log2-fold change in gene expression
D
Pathway
p-value
Mitotic cell cycle process 5.24E-35
Mitotic cell cycle 8.78E-30
Cell cycle process 8.46E-26
Cell cycle 1.03E-25
Cell division 2.65E-23
Chromosome segregation 4.84E-23
Mitotic cell cycle phase transition 9.93E-23
Cell cycle phase transition 2.98E-20
Nuclear chromosome segregation 8.57E-19
DNA metabolic process 2.75E-18
E
PLK1
0.015
Gene Expression (TPM)
Controls
RAS Wildtype
RAS Mutant
F
siNT
siNRAS
siNT
siKRAS
PLK1
β-actin
Control
NRASG12D
Control
KRASG12D
PLK1
β-actin
G
siNT, n=4
siJAK2 , n=4
6.44E-7
0.78
mRNA (relative expression)
JAK2
PLK1
siNT
siJAK2
JAK2
pSTAT3
PLK1
β-actin
H
siNT, n=4
siNRAS, n=4
2.96E-5
0.86
mRNA (relative expression)
NRAS
PLK1
siNT
siNRAS
pERK
tERK
PLK1
β-actin

Supplementary Figure 4. Mitotic kinase enrichment in pCMML is likely unrelated to bulk sequencing or *JAK2* mutation. A. Pearson's correlation comparing RNA-seq data presented as median log2-normalized transcript number. Measured transcripts from unsorted PB MNCs (n=5) were compared to transcripts from CD34+ sorted progenitor cells (left, n=5) as well as CD14+ sorted monocytes (right, n=5) in a pair-wise fashion. The indicated p-value is calculated by Spearman's rank correlation coefficient and hypothesis test. B. Heatmap comparing expression of housekeeping genes and select genes relevant to myeloid biology between CD34+ sorted and unsorted CMML patient-derived samples. C. Volcano plot comparing differential gene expression assessed by RNA-seq between CD34+ sorted (n=5) and unsorted (n=5) CMML patient-derived samples. D. Table illustrating the top ten cellular processes upregulated in pCMML relative to dCMML based on differential gene expression analysis. E. Expression of *PLK1* based on RNA-seq analysis of CD14+ sorted patient-derived cells from age-matched controls (n=10 biological replicates), *RAS* wildtype dCMML (n=4 biological replicates) and *RAS* mutant pCMML (n=6 biological replicates). Indicated p-value by two-tailed Student's t-test. F. Representative Western blots assessing the effect of PLK1 expression with knockdown of NRAS or KRAS in *NRAS/KRAS* mutant pCMML patient-derived MNCs (above). Blots also indicate effect of PLK1 expression with overexpression of $NRAS^{G12D}$ or $KRAS^{G12D}$ in *NRAS/KRAS* wildtype dCMML patient-derived MNCs (below). G. qPCR assessing *JAK2* and *PLK1* expression after siRNA depletion of JAK2 in *JAK2* mutant, *NRAS* wildtype pCMML patient-derived MNCs. H. qPCR assessing *NRAS* and *PLK1* expression after siRNA depletion of NRAS in *NRAS* mutant dCMML patient-derived MNCs. Western blots in panels F-H are representative of three experiments. Indicated p-values in panels G and H by two-tailed Student's t-test. Data in panels E, G and H are presented as mean ± SEM. The indicated n represents the number of biologic replicates. Source data are provided as a Source Data file.

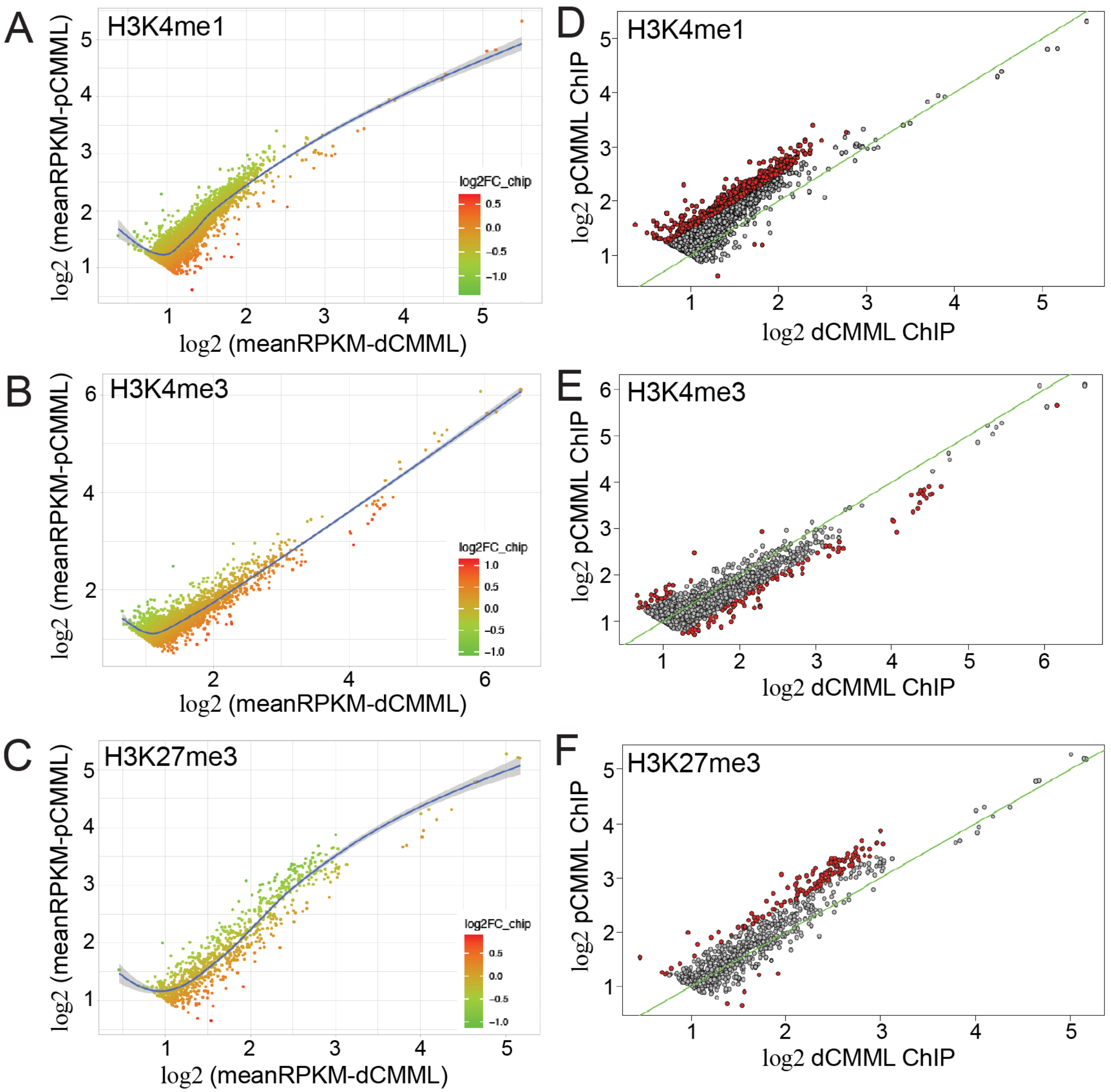

Supplementary Figure 5. H3K4me1 marks are preferentially enriched in pCMML relative to dCMML. Metagene analysis of epigenetic marks comparing dysplastic and proliferative samples. Scatterplot showing metagene analysis of genes showing relative enrichment for H3K4me1 (A), H3K4me3 (B), or H3K27me3 (C) aligned with RNA-seq data. RPKM represents reads per kilobase of transcript, per million mapped reads, a normalized unit of transcript expression. The color key histogram indicates ratio of epigenetic enrichment between proliferative and dysplastic samples. D-F. A similar analysis includes only enrichment comparisons between proliferative and dysplastic samples. Red points indicate differences in enrichment with log2 fold > 0.5 or < -0.5. The green line indicates predicted position based on no differences in enrichment between groups.

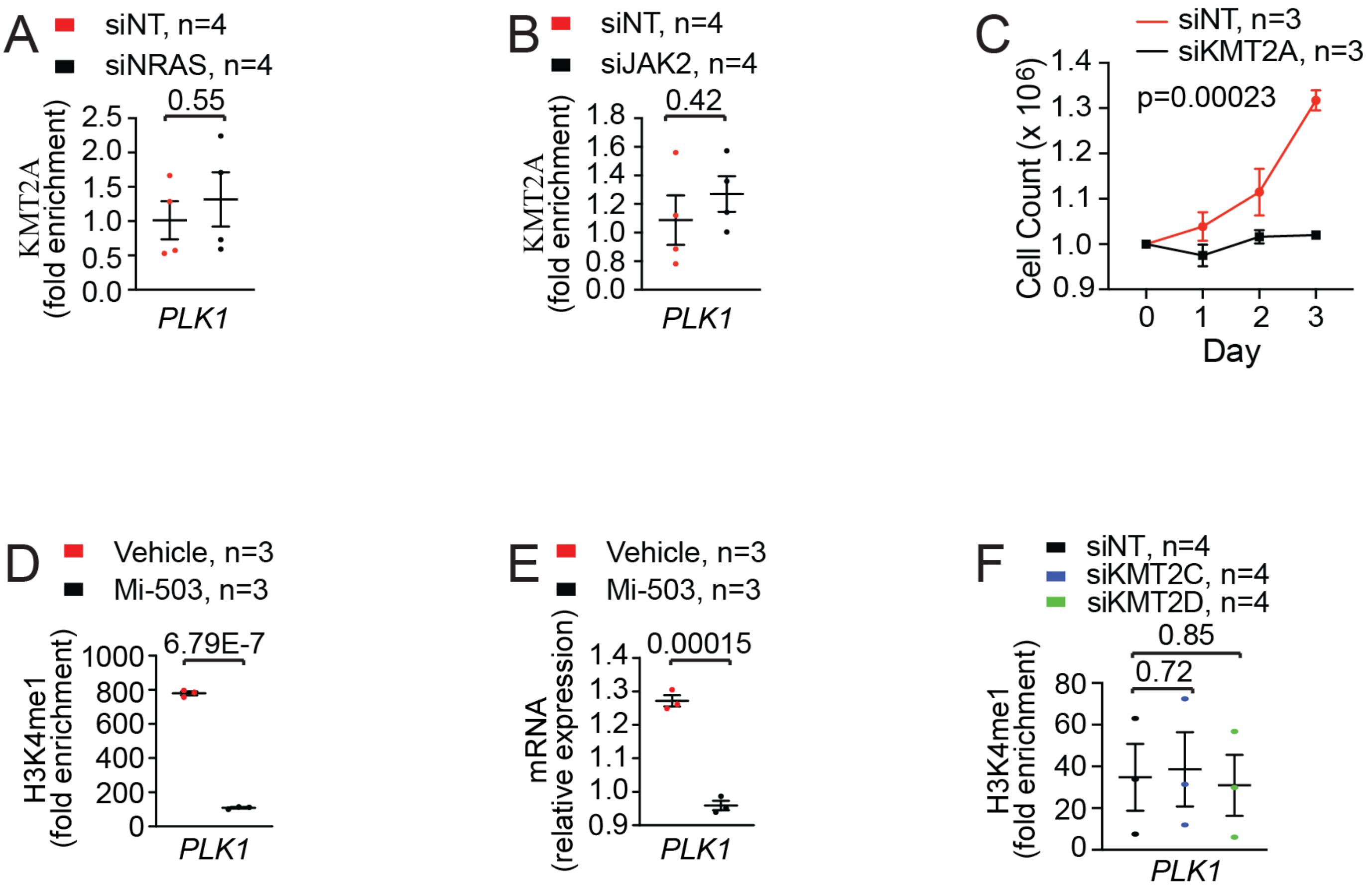

Supplementary Figure 6. KMT2A enrichment at the *PLK1* promoter is specific to *RAS* mutant pCMML. A. ChIP-PCR assessing KMT2A enrichment at the promoter of *PLK1* with (siNRAS) and without (siNT) knockdown of NRAS in *NRAS* mutant dCMML patient-derived MNCs. B. ChIP-PCR assessing KMT2A enrichment at the promoter of *PLK1* with (siJAK2) and without (siNT) knockdown of JAK2 in *JAK2* mutant, *NRAS* wildtype pCMML patient-derived MNCs. C. Daily cell counts of *NRAS* mutant pCMML patient-derived MNCs after siRNA depletion of KMT2A. D-E. ChIP-PCR assessing H3K4me1 enrichment at promoter of *PLK1* (D) and qPCR assessing PLK1 levels (E) in *NRAS* mutant pCMML patient-derived MNCs after treatment with either vehicle control or Mi-503. F. ChIP-PCR assessing occupancy of H3K4me1 at the promoter of *PLK1* after transfection of pCMML patient-derived MNCs with siNT, siKMT2C or siKMT2D. Data are presented as mean ± SEM. Indicated p-values in panels A-F by two-tailed Student's t-test. Source data are provided as a Source Data file.

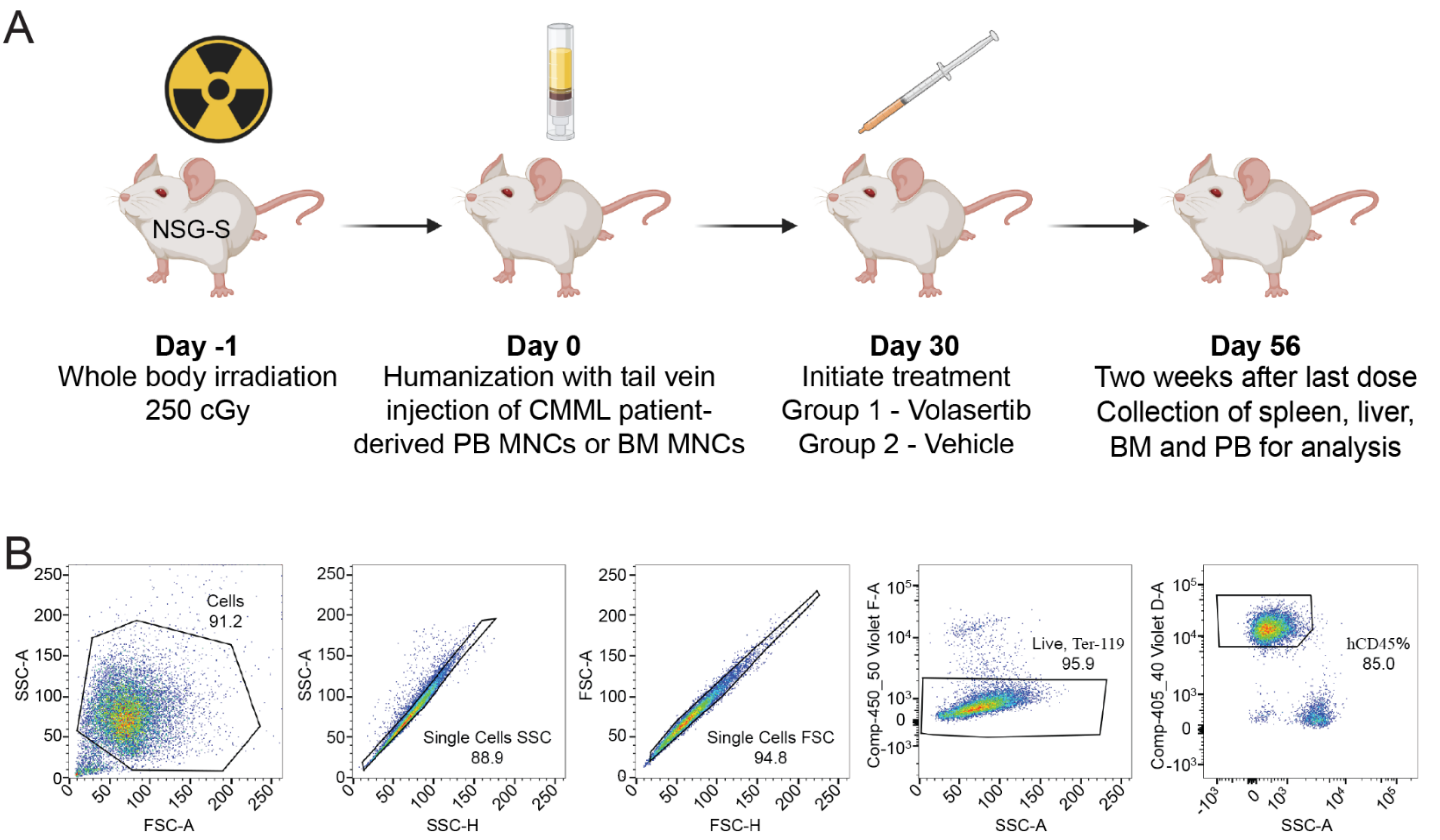

Supplementary Figure 7. Patient-derived xenograft drug study design. A. Diagram depicting the general experimental design of patient-derived xenograft (PDX) drug studies using NSG-S (NOD.Cg-*Prkdc*^{scid} *Il2rg*^{tm1Wjl}/SzJ-SGM3) mice. Peripheral blood (PB), Bone marrow (BM), Mononuclear cells (MNC). B. Flow cytometry gating strategy for PDX drug studies. SSC is side scatter. FSC is forward scatter. A and H indicate area and height respectively. Ter-119 is a lineage marker for erythroid cells. The hCD45 cells in the fifth panel was used for analyses seen in Figure 7E and F.

Supplementary Table 1. Clinical and laboratory features and subsequent events in 1183 WHO defined patients with chronic myelomonocytic leukemia (CMML) stratified by Mayo Clinic, Austrian and French (GFM) cohorts, Related to Figure 1

Variables [Median or n; range or %]	All patients (*n=1183*)	Mayo Clinic Cohort (*n*=397)	Austrian Cohort (*n*=175)	GFM Cohort (n=417)	P value
Age in years; median (range) Evaluable= 1181	72 (18.1-95.2)	70.6 (18.1-95.2)	72 (45-93)	73.64 (28.6-92.97)	0.0001
Sex (Male); *n* (%) Evaluable= 1165	774 (66)	397 (67)	106 (61)	271 (68)	0.21
Hemoglobin g/dL; median (range) Evaluable= 1162	11.1 (4.2-18)	10.7 (4.3-17)	11 (5.8-15.3)	11.7 (4.2-17.7)	0.0001
WBC x 10^9/L; median (range) Evaluable= 1183	12.6 (1.3-366.8)	12.9 (1.3-264.8)	17 (2.5-156)	11.3 (1.9-366.8)	0.0013
ANC x10^9/L; median (range) Evaluable= 576	6.2 (0-151)	6.2 (0-151)	NA	NA	-
AMC x 10^9/L; median (range) Evaluable= 1152	2.7 (1-102.5)	3 (1-84)	3.62 (1.015-54.4)	2.2 (1-102.5)	0.1
Platelets x 10^9/L; median(range) Evaluable= 1162	108 (3-1427)	101 (7-1277)	108 (5-726)	123 (3-1427)	0.0002
IMC (Y/N) Evaluable= 960	546 (57)	348 (60)	40 (73)	158 (49)	0.0005
PB blasts %; median (range) Evaluable= 1056	0 (0-19)	0 (0-19)	0 (0-17)	0 (0-14)	0.0002
BM blasts %; median (range) Evaluable= 941	4 (0-19)	3 (0-19)	NA	5 (0-19)	-
FAB CMML diagnosis Evaluable= 1183					
dCMML; *n* (%)	607 (51)	298 (50)	72 (41)	237 (57)	0.0019
pCMML; *n* (%)	576 (49)	293 (50)	103 (59)	180 (43)	
WHO 2016 CMML diagnosis Evaluable= 1065					
CMML-0; n (%)	530 (50)	323 (55)	75 (45)	132 (43)	0.0002
CMML-1; n (%)	304 (29)	158 (27)	41 (24)	105 (34)	
CMML-2; n (%)	231 (22)	106 (18)	52 (31)	73 (24)	
Mayo-French cytogenetic risk stratification; *n* (%) Evaluable= 1032					

Low	772 (75)	417 (74)	77 (66)	278 (79)	0.03
Intermediate	208 (20)	111 (20)	31 (27)	66 (19)	
High	52 (5)	34 (6)	8 (7)	10 (3)	
Next generation sequencing analysis; n (%) Evaluable=977					
1. Epigenetic regulators					
TET2	517 (53)	193 (50)	80 (46)	244 (60)	0.0015
IDH1	9 (1)	5 (1)	NA	4 (1)	-
IDH2	41 (5)	20 (5)	NA	21 (5)	-
DNMT3A	49 (6)	16 (4)	11 (6)	22 (7)	0.25
2. Chromatin regulators					
ASXL1	365 (37)	187 (48)	45 (26)	133 (32)	<0.0001
EZH2	54 (6)	16 (4)	18 (10)	20 (6)	0.017
3. Transcription factors					
RUNX1	76 (14)	49 (13)	27 (15)	NA	-
4. Spliceosome factors					
SRSF2	412 (44)	177 (46)	69 (39)	166 (44)	0.39
SF3B1	58 (6)	17 (4)	14 (8)	27 (7)	0.15
U2AF1	51 (7)	27 (7)	NA	24 (6)	-
ZRSR2	38 (5)	16 (4)	NA	22 (6)	-
5. Cell signaling					
NRAS	149 (15)	67 (17)	32 (18)	50 (12)	0.055
KRAS	87 (9)	20 (5)	16 (9)	51 (12)	0.002
CBL	145 (15)	67 (17)	28 (16)	50 (12)	0.10
PTPN11	26 (5)	14 (4)	12 (7)	NA	-
JAK2	73 (7)	25 (6)	17 (10)	31 (8)	0.39
CSF3R	29 (4)	4 (1)	NA	25 (7)	-
KIT	18 (3)	13 (3)	NA	5 (2)	0.13
MPL	3 (1)	3 (1)	NA	NA	-
CALR	0	0	NA	NA	-
6. Tumor suppressor gene					
Tp53	25 (3)	12 (3)	8 (5)	5 (2)	0.42
7. Others					
SETBP1	69 (9)	40 (10)	15 (9)	14 (8)	0.56
Mayo Molecular Model Evaluable= 743					
Low risk; n (%)	89 (12)	32 (8)	9 (17)	48 (15)	<0.0001
Intermediate-1 risk; n (%)	230 (31)	95 (25)	14 (26)	121 (39)	
Intermediate-2 risk; n (%)	231 (31)	131 (35)	14 (26)	86 (27)	
High risk; n (%)	193 (26)	119 (32)	16 (30)	58 (19)	
GFM Prognostic Model Evaluable= 938					
Low risk; n (%)	420 (45)	154 (40)	65 (37)	201 (53)	

Intermediate risk; n (%)	359 (38)	145 (40)	83 (48)	131 (34)	<0.0001
High risk; n (%)	159 (17)	85 (22)	26 (15)	48 (13)	
CPSS-Mol Prognostic Model<br>Evaluable= 339					
Low risk; n (%)	38 (11)	38 (11)	NA	NA	-
Intermediate-1 risk; n (%)	86 (25)	86 (25)	NA	NA	
Intermediate-2 risk; n (%)	131 (39)	131 (39)	NA	NA	
High risk; n (%)	84 (25)	84 (25)	NA	NA	
Deaths; *n* (%)<br>Evaluable= 995	652 (55)	394 (67)	112 (64)	146 (35)	<0.0001
Leukemic transformation; n (%)<br>Evaluable= 995	196 (20)	119 (20)	12 (7)	65 (28)	<0.0001

Key: CMML: chronic myelomonocytic leukemia, AMC: absolute monocyte count; ANC: absolute neutrophil count; IMC: immature circulating cells; WBC: white blood cell count; PB: peripheral blood; BM: bone marrow; WHO: World Health Organization; CPSS-Mol: clinical/molecular CMML-specific prognostic scoring system; dCMML: dysplastic chronic myelomonocytic leukemia; pCMML: proliferative chronic myelomonocytic leukemia; FAB: French-American-British; GFM: Groupe Francophone des Myelodyplasies; IMC: immature myeloid cells; ^{MT}: mutated; ^{WT}: wild type; CI: confidence interval ; NA: not available.

Supplementary Table 2. Clinical, pathological and molecular characteristics of 1183 WHO defined chronic myelomonocytic leukemia (CMML) patients stratified by proliferative and dysplastic subtypes at diagnosis, Related to Figure 1

Variables [Median or n; range or %]	All patients (*n=1183*)	pCMML (n=576)	dCMML (n=607)	P value
Age in years; median (range) Evaluable= 1181	72 (18.1-95.2)	71.34 (18.1-93)	73 (28.3-95.1)	0.004
Sex (Male); *n* (%) Evaluable= 1165	774 (66)	35 (63)	419 (70)	0.02
Hemoglobin g/dL; median (range) Evaluable= 1162	11.1 (4.2-18)	10.9 (4.2-17.7)	11.2 (4.6-16.8)	0.01
WBC x 10^9/L; median (range) Evaluable= 1183	12.6 (1.3-366.8)	24.1 (13-366.8)	7.2 (1.3-12.9)	<0.0001
ANC x10^9/L; median (range) Evaluable= 576	6.2 (0-151)	13.55 (1.5-151)	3.1 (0-11)	<0.0001
AMC x 10^9/L; median (range) Evaluable= 1152	2.5 (1-102.5)	4.7 (1-102.5)	1.5 (1-27.3)	<0.0001
Platelets x 10^9/L; median(range) Evaluable= 1162	108 (3-1427)	116 (5-1427)	104 (3-1051)	0.19
IMC (Y/N) Evaluable= 960	546 (57)	340 (74)	206 (41)	<0.0001
PB blasts %; median (range) Evaluable= 1056	0 (0-19)	0 (0-19)	0 (0-17)	<0.0001
BM blasts %; median (range) Evaluable= 941	4 (0-19)	4 (0-19)	4 (0-19)	0.41
WHO 2016 CMML diagnosis Evaluable= 1065				
CMML-0; n (%)	530 (50)	240 (46)	290 (54)	0.03
CMML-1; n (%)	304 (29)	159 (30)	145 (27)	
CMML-2; n (%)	231 (22)	126 (24)	105 (19)	
Mayo-French cytogenetic risk stratification; *n* (%) Evaluable= 1032				
Low	772 (75)	361 (72)	411 (77)	0.09
Intermediate	208 (20)	115 (23)	93 (18)	
High	52 (5)	25 (5)	27 (5)	

Next generation sequencing analysis; n (%)<br>Evaluable=977				
1. Epigenetic regulators				
TET2	517 (53)	227 (48)	290 (58)	0.0022
IDH1	9 (1)	3 (1)	6 (1)	0.44
IDH2	41 (5)	18 (5)	23 (5)	0.78
DNMT3A	49 (6)	24 (6)	25 (6)	0.93
2. Chromatin regulators				
ASXL1	365 (37)	221 (47)	144 (29)	<0.0001
EZH2	54 (6)	38 (9)	16 (3)	0.0011
3. Transcription factors				
RUNX1	76 (14)	36 (12)	40 (15)	0.36
4. Spliceosome factors				
SRSF2	412 (44)	202 (44)	210 (43)	0.8
SF3B1	58 (6)	22 (5)	36 (7)	0.1
U2AF1	51 (7)	25 (7)	26 (6)	0.63
ZRSR2	38 (5)	9 (3)	29 (7)	0.0041
5. Cell signaling				
NRAS	149 (15)	110 (23)	39 (8)	<0.0001
KRAS	87 (9)	43 (9)	44 (9)	0.84
CBL	145 (15)	85 (18)	60 (12)	0.009
PTPN11	26 (5)	14 (5)	12 (5)	0.9
JAK2	73 (7)	51 (11)	22 (4)	0.0002
CSF3R	29 (4)	15 (4)	14 (4)	0.58
KIT	18 (3)	11 (3)	7 (2)	0.24
MPL	3 (1)	2 (1)	1 (1)	0.55
CALR	0	0	0	-
6. Tumor suppressor gene				
Tp53	25 (3)	12 (3)	13 (3)	0.78
7. Others				
SETBP1	69 (9)	42 (11)	27 (8)	0.1
Mayo Molecular Model<br>Evaluable= 743				
Low risk; n (%)	89 (12)	23 (7)	66 (17)	<0.0001
Intermediate-1 risk; n (%)	230 (31)	82 (23)	148 (38)	
Intermediate-2 risk; n (%)	231 (31)	113 (32)	118 (30)	
High risk; n (%)	193 (26)	135 (38)	58 (15)	
GFM Prognostic Model<br>Evaluable= 938				
Low risk; n (%)	420 (45)	62 (14)	358 (74)	<0.0001
Intermediate risk; n (%)	359 (38)	255 (56)	104 (21)	
High risk; n (%)	159 (17)	135 (30)	24 (5)	
CPSS-Mol Prognostic Model<br>Evaluable= 339				

Low risk; n (%)	38 (11)	0	38 (22)	<0.0001
Intermediate-1 risk; n (%)	86 (25)	25 (15)	61 (35)	
Intermediate-2 risk; n (%)	131 (39)	76 (46)	55 (32)	
High risk; n (%)	84 (25)	65 (39)	19 (11)	
Deaths; *n* (%)<br>Evaluable= 995	652 (55)	346 (60)	306 (50)	0.0008
Leukemic transformation;<br>n (%)<br>Evaluable= 995	196 (20)	99 (20)	97 (19)	0.53

Key: CMML: chronic myelomonocytic leukemia, AMC: absolute monocyte count; ANC: absolute neutrophil count; IMC: immature circulating cells; WBC: white blood cell count; PB: peripheral blood; BM: bone marrow; WHO: World Health Organization; CPSS-Mol: clinical/molecular CMML-specific prognostic scoring system; dCMML: dysplastic chronic myelomonocytic leukemia; pCMML: proliferative chronic myelomonocytic leukemia; FAB: French-American-British; GFM: Groupe Francophone des Myelodyplasies; IMC: immature myeloid cells; ^{MT}: mutated; ^{WT}: wild type; CI: confidence interval

Supplementary Table 3. Clinical and pathological characteristics of CMML patients tested through a research-based whole exome sequencing (WES) platform, Related to Figure 2

Variables [Median or n; range or %]	*CMML WES cohort (n=48)*
Age in years; median (range)	69.55 (18.04-86.82)
Sex (Male); *n* (%)	28 (58)
Hemoglobin g/dL; median (range)	10.85 (4.3-16)
WBC x 10^9/L; median (range)	13.8 (2.8-264.8)
ANC x10^9/L; median (range)	83.25 (0.84-37839.92)
AMC x 10^9/L; median (range)	2.8 (1-24.8)
Platelets x 10^9/L; median (range)	98.5 (14-362)
IMC (Y/N) *n* (%)	30 (63)
PB blasts %; median (range)	0 (0-19)
BM blasts %; median (range)	4 (0-18)
FAB CMML diagnosis	
dCMML; *n* (%)	22 (46)
pCMML; *n* (%)	26 (54)
WHO 2016 CMML diagnosis	
CMML-0; n (%)	18 (38)
CMML-1; n (%)	17 (35)
CMML-2; n (%)	13 (27)
Mayo-French cytogenetic risk stratification; *n* (%)	
Low	27 (56)
Intermediate	9 (19)
High	6 (13)
Mayo Molecular Model	
Low risk; n (%)	3 (63)
Intermediate-1 risk; n (%)	10 (21)
Intermediate-2 risk; n (%)	4 (8)
High risk; n (%)	11 (23)
GFM Prognostic Model	

Low risk; n (%)	10 (21)
Intermediate risk; n (%)	12 (25)
High risk; n (%)	7 (15)
CPSS-Mol Prognostic Model	
Low risk; n (%)	3 (63)
Intermediate-1 risk; n (%)	8 (17)
Intermediate-2 risk; n (%)	3 (63)
High risk; n (%)	12 (25)
Deaths; *n* (%)	38 (80)
Leukemic transformation; n (%)	48 (100)

Key: CMML: chronic myelomonocytic leukemia, WES: whole exome sequencing, AMC: absolute monocyte count; ANC: absolute neutrophil count; IMC: immature circulating cells; WBC: white blood cell count; PB: peripheral blood; BM: bone marrow; WHO: World Health Organization; CPSS-Mol: clinical/molecular CMML-specific prognostic scoring system; dCMML: dysplastic chronic myelomonocytic leukemia; pCMML: proliferative chronic myelomonocytic leukemia; FAB: French-American-British; GFM: Groupe Francophone des Myelodyplasies; IMC: immature myeloid cells; ^{MT}: mutated; ^{WT}: wild type; CI: confidence interval

Supplementary Table 4. Primer sets and their sequences

RT-PCR Primer Name	*Primer Sequence*	
GAPDH	Sense	GACCTGACCTGCCGTCTAGAAAAA
	Antisense	ACCACCCTGTTGCTGTAGCCAAAT
JAK2	Sense	TATGATGAGCAAGCTTTCTCACAAG
	Antisense	TCCAAATTTACAAACTCCTGAACC
KMT2A	Sense	ACATCGTCAGCCTCCTGAATACA
	Antisense	ACACCAACTGCCTCCTTAGAA
KRAS	Sense	CAAGAGTGCCTTGACGATACA
	Antisense	GACCTGCTGTGTCGAGAATATC
NRAS	Sense	ACCTCTACAGGGAGCAGATTA
	Antisense	CTGTCCTTGTTGGCAAATCAC
PLK1	Sense	AGAAAGGGCACAGTTTCGAG
	Antisense	GGGTTGATGTGCTTGGGAATA
TBP	Sense	TATAATCCCAAGCGGTTTGC
	Antisense	CCCAACTTCTGTACAACTCTAGCA
ChIP Primer Name	*Primer Sequence*	
PLK1 promoter	Sense	CTATGACCTGCCAGTTTGCTA
	Antisense	GCCTCCTCGCTACTGAATTT

Key: GAPDH: glyceraldehyde 3-phosphate dehydrogenase, JAK2: janus kinase 2, KMT2A: lysine methyltransferase 2A, PLK1: polo-like kinase 1, TBP: TATA-binding protein, RT-PCR: reverse transcription polymerase chain reaction, ChIP: chromatin immunoprecipitation
