# Supplementary material for: RAS mutations drive proliferative chronic myelomonocytic leukemia via a KMT2A-PLK1 axis
Source: Nat Commun. 2021 May 18;12:2901. doi: 10.1038/s41467-021-23186-w (PMC8131698; doi:10.1038/s41467-021-23186-w)
Supplement: Supplementary file 3 — Description of Additional Supplementary Files [file 41467_2021_23186_MOESM3_ESM.pdf]

Description of Additional Supplementary Files

File Name: Supplementary Data 1
Description: RNA-sequencing data depicting individual gene results. Each row shows a given gene's ENSEMBL identification number, gene name, logarithmic function of the fold change in gene expression (Log2FC) in pCMML relative to dCMML, and false discovery rate (FDR).

File Name: Supplementary Data 2
Description: Data set demonstrating the overlap between H3K4me1 enrichment by ChIP-seq and overexpression by RNA-seq. The first column lists genes that were identified to be uniquely H3K4me1 enriched in pCMML relative to dCMML by ChIP-seq. Column two lists genes overexpressed in pCMML relative to dCMML by RNA-seq. The associated logarithmic function of the fold change in gene expression (Log2FC) in pCMML relative to dCMML, and false discovery rate (FDR) are in columns three and four respectively. The list of genes both uniquely H3K4me1 enriched and overexpressed in pCMML relative to dCMML is found in column five.
