# Supplementary material for: RAS mutations drive proliferative chronic myelomonocytic leukemia via a KMT2A-PLK1 axis
Source: Nat Commun. 2021 May 18;12:2901. doi: 10.1038/s41467-021-23186-w (PMC8131698; doi:10.1038/s41467-021-23186-w)
Supplement: Supplementary file 6 — Reporting Summary [file 41467_2021_23186_MOESM6_ESM.pdf]

nature research

Corresponding author(s): Sergey Nikolaev and Mrinal M. Patnaik

Last updated by author(s): 4/15/2021

Reporting Summary

Nature Research wishes to improve the reproducibility of the work that we publish. This form provides structure for consistency and transparency in reporting. For further information on Nature Research policies, see our Editorial Policies and the Editorial Policy Checklist.

Statistics

For all statistical analyses, confirm that the following items are present in the figure legend, table legend, main text, or Methods section.

n/a	Confirmed	
☐	☒	The exact sample size (*n*) for each experimental group/condition, given as a discrete number and unit of measurement
☐	☒	A statement on whether measurements were taken from distinct samples or whether the same sample was measured repeatedly
☐	☒	The statistical test(s) used AND whether they are one- or two-sided<br>*Only common tests should be described solely by name; describe more complex techniques in the Methods section.*
☐	☒	A description of all covariates tested
☐	☒	A description of any assumptions or corrections, such as tests of normality and adjustment for multiple comparisons
☐	☒	A full description of the statistical parameters including central tendency (e.g. means) or other basic estimates (e.g. regression coefficient) AND variation (e.g. standard deviation) or associated estimates of uncertainty (e.g. confidence intervals)
☐	☒	For null hypothesis testing, the test statistic (e.g. *F*, *t*, *r*) with confidence intervals, effect sizes, degrees of freedom and *P* value noted<br>*Give P values as exact values whenever suitable.*
☒	☐	For Bayesian analysis, information on the choice of priors and Markov chain Monte Carlo settings
☒	☐	For hierarchical and complex designs, identification of the appropriate level for tests and full reporting of outcomes
☐	☒	Estimates of effect sizes (e.g. Cohen's *d*, Pearson's *r*), indicating how they were calculated

Our web collection on statistics for biologists contains articles on many of the points above.

Software and code

Policy information about availability of computer code

Data collection	Burrows-Wheeler Aligner, MAPRSeq 2.0, TopHat 2.06, HISAT2 2.2.1, bowtie2 2.4.2, samtools 1.11, BD FACSDiva 9.0
Data analysis	Agilent SureCall 4.2, MuTect 4.1.0.0, FACETS 0.5.0, ClonEvol, Genome Analysis Toolkit 4.1.2.0, Pandas 1.2.3, featureCounts 2.0.0, edgeR 3.12, heatmap.2, DESeq2 1.30.1, pheatmap 1.0.12, rgl 0.105.22, MACS2 2.2.6.1, Homer mergePeaks 4.11.1, DiffBind 2.16.0, R 4.0.3, deeptools 2.0

For manuscripts utilizing custom algorithms or software that are central to the research but not yet described in published literature, software must be made available to editors and reviewers. We strongly encourage code deposition in a community repository (e.g. GitHub). See the Nature Research guidelines for submitting code & software for further information.

Data

Policy information about availability of data

All manuscripts must include a data availability statement. This statement should provide the following information, where applicable:

- Accession codes, unique identifiers, or web links for publicly available datasets
- A list of figures that have associated raw data
- A description of any restrictions on data availability

WES data that support the findings in Figure 2 have been deposited in EGA under ID EGAD00001007026 [https://ega-archive.org/datasets/EGAD00001007026]. RNA-seq data that support the findings in Figures 4 and 5 have been deposited in GEO under series GSE156209 [https://www.ncbi.nlm.nih.gov/geo/query/acc.cgi?acc=GSE156209]. The ChIP-seq data that support the findings of this study have been deposited in GEO under series GSE156377 [https://www.ncbi.nlm.nih.gov/geo/query/acc.cgi?acc=GSE156377]. The UCSC session can be accessed through the genome browser [https://genome.ucsc.edu/s/mbinder/CMML_RAS_H3K4me1]. Source data are provided with this paper.

Field-specific reporting

Please select the one below that is the best fit for your research. If you are not sure, read the appropriate sections before making your selection.

[x] Life sciences [ ] Behavioural & social sciences [ ] Ecological, evolutionary & environmental sciences

For a reference copy of the document with all sections, see nature.com/documents/nr-reporting-summary-flat.pdf

Life sciences study design

All studies must disclose on these points even when the disclosure is negative.

Sample size	This was a retrospective assessment of a very large conglomerate of CMML patients. Given the rarity of this disease, and the retrospective nature of clinical/genetic correlates, sample size calculation was not possible.
Data exclusions	No data was excluded.
Replication	Each experiment was replicated at least three times. Number of biological replications are indicated in the associated figures. All attempts at replication were successful.
Randomization	Randomization was not applicable to the current study. Experiments were controlled using empty vectors when over-expressing genes or non-targeted RNA when performing knockdown experiments. Furthermore, a diversity of samples were selected and controlled by the presence or absence of driver mutations of interest. PDX treatment studies were conducted as a single-arm, proof-of-concept study with each engrafted genotype treated with both the control and experimental treatment.
Blinding	Investigators treating PDX mice with either vehicle or volasertib were blinded to the genotype of the engrafted patient's disease. Blinding was otherwise applicable with other experiments as experimental variables required knowledge of patient cell genotypes.

Reporting for specific materials, systems and methods

We require information from authors about some types of materials, experimental systems and methods used in many studies. Here, indicate whether each material, system or method listed is relevant to your study. If you are not sure if a list item applies to your research, read the appropriate section before selecting a response.

Materials & experimental systems

n/a	Involved in the study	
[ ]	[x]	Antibodies
[x]	[ ]	Eukaryotic cell lines
[x]	[ ]	Palaeontology and archaeology
[ ]	[x]	Animals and other organisms
[ ]	[x]	Human research participants
[x]	[ ]	Clinical data
[x]	[ ]	Dual use research of concern

Methods

n/a	Involved in the study	
[ ]	[x]	ChIP-seq
[ ]	[x]	Flow cytometry
[x]	[ ]	MRI-based neuroimaging

Antibodies

Antibodies used

Antibody Target (Supplier, Catalog Number, Clone)
PLK1 (Cell Signaling, 4535)
KMT2A (Cell Signaling, 14197, D6G8N)
phospho-ERK1/2 (Thr202/Tyr204) (Cell Signaling, 4370, D13.14.4E)
JAK2 (Cell Signaling, 3230, D2E12)
phospho-STAT3 (Cell Signaling, 9145, D3A7)
Vinculin (Bethyl, A302-535A)
Beta-actin (Cell Signaling, 4970, 13E5)
Total ERK1/2 (R&D, MAB1576)
NRAS (Santa Cruz, Sc-31, F155)
HRP-conjugated goat anti-rabbit IgG secondary antibody (Millipore, 12-348)
HRP-conjugated goat anti-mouse IgG secondary antibody (Millipore, 12-349)

KMT2A (Abcam, ab272023)
H3K4me1 (Abcam, ab176877, ERP16597)
H3K4me3 (Abcam, ab213224, EPR20551-225)
H3K27me3 (Abcam, ab192985, EPR18607)
Normal Rabbit IgG (EMD Millipore, 12-370)

	Normal Mouse IgG (EMD Millipore, 12-371)<br><br>Mac-1 (eBioscience, 17-0112-82, M1/70)<br>Gr-1 (eBioscience, 14-5931-82, RB6-8C5)<br>B220 (eBiosciences, 14,0452-82, RA3-6B2)<br>CD3 (Biolegend, 100201, 17A2)<br>CD4 (eBiosciences, 14-0041-82, GK1.5)<br>CD8 (eBiosciences, 14-0081-82, 53-6.7)<br>TERR119 (eBiosciences, 14-5921-82, TER-119)<br>CD117/c-Kit (eBiosciences, 17-1171-82, 2B8)<br>APC-conjugated donkey anti-rabbit F(ab')2 fragment (Jackson ImmunoResearch, AB-2340601)<br>Murine CD45.1 BUV737 (BD Biosciences, 564574, A20)<br>Human CD45 BV605 (BD Biosciences, 564048)<br>Human CD3 APC (BD Biosciences, 561810, UCHT1)<br>Human CD33 PE (BD Biosciences, 555450, WM53)<br>CD3 (Abcam, ab17143, F7.2.38)<br>CD14 (Sigma, HPA001887)<br>CD163 (Leica, PA0090, 10D6)
Validation	Antibody Target (Species Source; Species Reactivity; Application Validation)<br>PLK1 (Rabbit; Human, Mouse, Rabbit Monkey; WB at 1:500)<br>KMT2A (Rabbit; Human, Mouse, Rabbit, Monkey; WB at 1:1000, IP at 1:50, IF at 1:200)<br>phospho-ERK1/2 (Thr202/Tyr204) (Rabbit; Human, Mouse, Rabbit Human, Monkey; WB at 1:2000, IP at 1:40; IHC at 1:200-1:800; IF at 1:200-1:400, flow cytometry at 1:800-1:1600)<br>JAK2 (Rabbit; Human, Mouse, Rabbit; WB at 1:1000, IP at 1:100, IHC at 1:800-1:3200)<br>phospho-STAT3 (Rabbit; Human, Mouse, Rabbit, Monkey; WB at 1:2000, IP at 1:100, IHC at 1:100-1:400, IF at 1:100-1:200, flow cytometry at 1:100-1:400, Chromatin IP at 1:100, Chromatin IP-seq at 1:100)<br>Vinculin (Rabbit; Human, Mouse; WB at 1:2000-1:10000, IP at 5-15 ug/mg lysate, IHC at 1:500-1:2000)<br>Beta-actin (Rabbit; Human, Mouse, Rabbit, Monkey; WB at 1:1000, IHC at 1:50-1:200, IF at 1:100-1:400, flow cytometry at 1:100-1:400)<br>Total ERK1/2 (Mouse; Human, Mouse, Rat; WB at 0.5 ug/mL, IHC at 8-25 ug/mL)<br>NRAS (Mouse; Mouse, Rat, Human; WB at 1:100 to 1:1000, IP at 1-2 ug/mL lysate, IF at 1:50 to 1:500, IHC at 1:50-1:500)<br><br>KMT2A (Rabbit; Human; WB at 1:2000-1:10000, IP at 2-5 ug/mg lysate, ChIP at 10 ug)<br>H3K4me1 (Rabbit; Mouse, Rat, Human; WB at 1:5000, IF at 1:1000, IHC at 1:1000, ChIP at 2 uL)<br>H3K4me3 (Rabbit; Mouse, Rat, Human; ChIP-seq at 4 ug for 30 ug chromatin, ChIP at 2 ug for 25 ug of chromatin, flow cytometry at 1:500, WB at 1:1000, IF at 1:500, IP at 1:30)<br>H3K27me3 (Rabbit; Mouse, Rate, Human; ChIP-seq at 4 ug for 10^7 cells, ChIP at 2 ug for 25 ug of chromatin, IF at 1:1000, IHC at 1:500, WB at 1:1000)<br>Normal Rabbit IgG (Rabbit; Human, Mouse, Rat; ChIP at 1:50)<br>Normal Mouse IgG (Mouse; Human, Mouse, Rat; ChIP at 1:50)<br><br>Mac-1 (Rat; Mouse; Flow cytometry at 0.125 ug/test)<br>Gr-1 (Rat, Mouse, Flow cytometry at 0.5 ug/test)<br>B220 (Rat; Mouse, Human; Flow cytometry at 1 ug/test; IHC at 5 ug/mL)<br>CD3 (Rat; Mouse; Flow cytometry at 0.25 ug/test, IHC at 10 ug/mL)<br>CD4 (Rat; Mouse; Flow cytometry at 0.125 ug/test)<br>CD8 (Rat; Mouse; Flow cytometry at 0.25 ug/test)<br>TERR119 (Rat; Mouse; Flow cytometry at 0.5 ug/test)<br>CD117/c-Kit (Rat; Mouse, Pig; Flow cytometry at 0.125 ug/test)<br>Murine CD45.1 BUV737 (Mouse; Mouse; Flow cytometry)<br>Human CD45 BV605 (Mouse; Human; Flow cytometry)<br>Human CD3 APC (Mouse; Human; Flow cytometry at 5uL/test)<br>Human CD33 PE (Mouse; Human; Flow cytometry at 1:50)<br>CD3 (Mouse; Human; IHC at 1:10-1:25)<br>CD14 (Rabbit; Human; WB at 0.04-0.4 ug/mL, IF at 0.25-2 ug/mL, IHC at 1:200-1:500)<br>CD163 (Mouse; Human; IHC at 1:200)

Animals and other organisms

Policy information about studies involving animals; ARRIVE guidelines recommended for reporting animal research

Laboratory animals	The NOD.Cg-Prkdc scid Il2rg tm1Wjl / S J-SGM3 (NSGS) mice were used for the PDX experiments. Both male and female mice were used. The genetically engineered mouse model was bred on a C57BL/6 background. Both male and female mice were used. The mice were 6 to 8 weeks old at the start of the experiments.
Wild animals	The study did not involve wild animals.
Field-collected samples	The study did not involve field-collected samples.

Ethics oversight	Mayo Clinic IACUC (protocol A00003488-18)<br>Moffitt Cancer Center in Florida IACUC (protocol 6041)<br>University of Wisconsin at Madison IACUC (protocol M005328)

Note that full information on the approval of the study protocol must also be provided in the manuscript.

Human research participants

Policy information about studies involving human research participants

Population characteristics	This has been extensively and thoroughly reported in Supplementary Tables 1-3 in the manuscript and will not be replicated here. Please refer to the Supplementary Information document submitted.
Recruitment	The CMML patients were accrued at individual centers based upon existing IRB approved protocols. At Mayo Clinic, the IRB protocol allows patients being seen at the clinic for their CMML diagnosis to consent to biospecimen collection/banking. The bias that could set in with this process is that patients who are symptomatic from their disease are more likely to seek care and, hence, higher risk patients could be more enriched in this dataset.
Ethics oversight	Mayo Clinic Institutional Review Board (IRB-15-003786)<br>Gustave Roussy Cancer Center ethical committee Ile-de-France 1 (DC-2014-2091)<br>Ethics committee of the City of Vienna (15-059-VK)

Note that full information on the approval of the study protocol must also be provided in the manuscript.

ChIP-seq

Data deposition

☒ Confirm that both raw and final processed data have been deposited in a public database such as GEO.

☒ Confirm that you have deposited or provided access to graph files (e.g. BED files) for the called peaks.

Data access links<br>*May remain private before publication.*	GSE156377: https://www.ncbi.nlm.nih.gov/geo/query/acc.cgi?acc=GSE156377
Files in database submission	GSE156377 Methylation and chromatin abnormalities in chronic myelomonocytic leukemia. Aug 18, 2020 approved None<br>GSM4729826 01MMDY Aug 18, 2020 approved TXT BED TXT BED<br>TXT BED<br>GSM4729827 02MMDN Aug 18, 2020 approved TXT BED TXT BED<br>TXT BED<br>GSM4729828 03MMDY Aug 18, 2020 approved TXT BED TXT BED<br>TXT BED<br>GSM4729829 04MMDN Aug 18, 2020 approved TXT BED TXT BED<br>TXT BED<br>GSM4729830 06MMDN Aug 18, 2020 approved TXT BED TXT BED<br>TXT BED<br>GSM4729831 07FMDY Aug 18, 2020 approved TXT BED TXT BED<br>TXT BED<br>GSM4729832 08MMDN Aug 18, 2020 approved TXT BED TXT BED<br>TXT BED<br>GSM4729833 10FMPY Aug 18, 2020 approved TXT BED TXT BED<br>TXT BED<br>GSM4729834 11MMPN Aug 18, 2020 approved TXT BED TXT BED<br>TXT BED<br>GSM4729835 12FMPN Aug 18, 2020 approved TXT BED TXT BED<br>TXT BED<br>GSM4729836 13MMPN Aug 18, 2020 approved TXT BED TXT BED<br>TXT BED<br>GSM4729837 14FMPN Aug 18, 2020 approved TXT BED TXT BED<br>TXT BED<br>GSM4729838 15FMPN Aug 18, 2020 approved TXT BED TXT BED<br>TXT BED<br>GSM4729839 16FMPY Aug 18, 2020 approved TXT BED TXT BED<br>TXT BED<br>GSM4729840 17MMPY Aug 18, 2020 approved TXT BED TXT BED<br>TXT BED<br>GSM4729841 18MMPN Aug 18, 2020 approved TXT BED TXT BED<br>TXT BED

Genome browser session (e.g. UCSC) | https://genome.ucsc.edu/s/mbinder/CMML_RAS_H3K4me1

Methodology

Replicates	7 proliferative and 9 dysplastic bone marrow samples.
Sequencing depth	Sequencing was on an Illumina HiSeq2500 sequencer to an average depth of 26,582,952 for H3K4me1, 28,117,131 for H3K4me3, 58,105,538 for H3K27me3, and 65,675,440 for Input per sample.
Antibodies	H3K4me1 (Diagenode, C15410037), H3K3me3 (Cell Signaling Technology, 9751S), H3K27me3 (Diagenode, pAb-069-050)
Peak calling parameters	Peaks were called using MACS2. All peaks were called relative to their respective input controls using the paired end mode at a default q-value cut-off of <0.05. Only peaks replicated (in >55% of samples (5/9 and 4/7)) were used in downstream analyses.
Data quality	Read data was assessed with fastqc (http://www.bioinformatics.babraham.ac.uk/projects/fastqc/) to determine read quality and integrity. Duplicate reads were removed prior to alignment; truncated, unpaired and chimeric reads were discarded from any downstream analyses post-alignment. Overall data quality was quite high, with low duplication levels (<8% average overall) and high alignment rates (>96% overall average in all samples).
Software	MACS2, DiffBind, Homer, Bowtie2, samtools, and deeptools (all mentioned in the methods)

Flow Cytometry

Plots

Confirm that:

- [x] The axis labels state the marker and fluorochrome used (e.g. CD4-FITC).
- [x] The axis scales are clearly visible. Include numbers along axes only for bottom left plot of group (a 'group' is an analysis of identical markers).
- [x] All plots are contour plots with outliers or pseudocolor plots.
- [x] A numerical value for number of cells or percentage (with statistics) is provided.

Methodology

Sample preparation	Cells of PDX experiments were collected from blood, BM and spleen and stained with the following antibodies: murine CD45.1 BUV737 (BD Biosciences-564574), human CD45 BV605 (BD Biosciences-564048), human CD3 APC (BD Biosciences-561810) and human CD33 PE (BD Biosciences-555450).
Instrument	LSRII flow cytometer (BD Biosciences).
Software	BD FACSDiva software for the BD LSRII flow cytometer.
Cell population abundance	As outlined in supplementary figure 7B, single cells were gated for from indicated homogenized tissues using forward and side scatter. Fixable viability marker, Zombie Violet, with TER-119 were used to specifically select for live cells. Finally, a minimum of 100,000 events was obtained to preformed the indicated analyses in Figure 7.
Gating strategy	We used a fixable viability marker (Zombie Violet from Biolegend) and TER-119 (V450 from BD Biosciences) on the same channel to exclude dead cells and erythrocytes before gating the hCD45 and mCD45.1 cells.

- [x] Tick this box to confirm that a figure exemplifying the gating strategy is provided in the Supplementary Information.
